# Correction to: Evaluation of admission levels of P, E and L selectins as predictors for thrombosis in hospitalized COVID-19 patients

**DOI:** 10.1007/s10238-022-00802-7

**Published:** 2022-02-16

**Authors:** Mona M. Watany, Said Abdou, Reham Elkolaly, Nashwa Elgharbawy, Hossam Hodeib

**Affiliations:** 1 Clinical Pathology Department, Faculty of Medicine, Tanta University, Medical Campus, El Giesh st., Tanta, El-Gharbia Governorate, Tanta, 31527 Egypt; 2 Chest department, Faculty of Medicine, Tanta University, Tanta, Egypt; 3 Internal Medicine Department, Faculty of Medicine, Tanta University, Tanta, Egypt

## Correction to: Clinical and Experimental Medicine 10.1007/s10238-021-00787-9

The original version of this article unfortunately contained a mistake. Author name Said Abdou was incorrectly written as Saied Abdou.

